# The Genomic Impact of DNA CpG Methylation on Gene Expression; Relationships in Prostate Cancer

**DOI:** 10.3390/biom7010015

**Published:** 2017-02-14

**Authors:** Mark D. Long, Dominic J. Smiraglia, Moray J. Campbell

**Affiliations:** 1 Department of Cancer Genetics, Roswell Park Cancer Institute, Buffalo, NY 14263, USA; Mark.long@roswellpark.org (M.D.L.); dominic.smiraglia@roswellpark.org (D.J.S.); 2 College of Pharmacy, Pharmaceutics and Pharmaceutical Chemistry, 536 Parks Hall, 500 West 12th Ave., The Ohio State University, Columbus, OH 43210, USA

**Keywords:** CpG methylation, gene expression, genome-wide, prostate cancer, Indolethylamine *N*-Methyltransferase, Methionine Adenosyltransferase 2B

## Abstract

The process of DNA CpG methylation has been extensively investigated for over 50 years and revealed associations between changing methylation status of CpG islands and gene expression. As a result, DNA CpG methylation is implicated in the control of gene expression in developmental and homeostasis processes, as well as being a cancer-driver mechanism. The development of genome-wide technologies and sophisticated statistical analytical approaches has ushered in an era of widespread analyses, for example in the cancer arena, of the relationships between altered DNA CpG methylation, gene expression, and tumor status. The remarkable increase in the volume of such genomic data, for example, through investigators from the Cancer Genome Atlas (TCGA), has allowed dissection of the relationships between DNA CpG methylation density and distribution, gene expression, and tumor outcome. In this manner, it is now possible to test that the genome-wide correlations are measurable between changes in DNA CpG methylation and gene expression. Perhaps surprisingly is that these associations can only be detected for hundreds, but not thousands, of genes, and the direction of the correlations are both positive and negative. This, perhaps, suggests that CpG methylation events in cancer systems can act as disease drivers but the effects are possibly more restricted than suspected. Additionally, the positive and negative correlations suggest direct and indirect events and an incomplete understanding. Within the prostate cancer TCGA cohort, we examined the relationships between expression of genes that control DNA methylation, known targets of DNA methylation and tumor status. This revealed that genes that control the synthesis of *S*-adenosyl-l-methionine (SAM) associate with altered expression of DNA methylation targets in a subset of aggressive tumors.

## 1. Introduction

### 1.1. Methylation of DNA Cytosine

The patterns and function of DNA methylation have been extensively investigated across organisms, in settings of both health and disease (reviewed in [1]). In humans, for example, this covalent DNA modification has been studied in developmental and normal biology [2] where it is clear that the control of DNA methylation is biologically profound. The control of DNA methylation is central to embryogenesis, genetic imprinting, and X chromosome inactivation. Furthermore, DNA methylation levels change across the genome through the aging process, thus giving rise to the concept of the so-called epigenetic clock (reviewed in [3,4]). There has been an equally profound examination of DNA methylation in cancer settings (reviewed in [5]) and other age-related syndromes.

At the center of the process of DNA methylation is the transfer of a methyl group from *S*-adenosyl-l-methionine (SAM) to the cytosine of a CpG dinucleotide (adjacent within a single DNA strand), immediately following DNA replication [6]. The addition of a methyl group to cytosine most commonly occurs in the context of a being adjacent and five prime to guanine and, hence, the nomenclature of CpG where p represents the DNA phosphate backbone. Cytosine can also be hydroxyl methylated.

In fact, SAM is a universal methyl donor, being the substrate for enzymes that control the methylated status of DNA, RNA [7], and proteins, such as histones [8]. Indeed, varieties of the enzymes that control these events have relatively high affinity for SAM [9]. Following donation of a methyl group, *S*-adenosyl-l-homocysteine (SAH) is formed and the ratio of SAM/SAH appears to be critical for the control of these biological processes (reviewed in [10]). Importantly, these substrates are, themselves, profoundly and rapidly influenced by environmental factors including diet [11] and, in turn this potentially combines with genetic variation and links to the predisposition to a number of diseases (reviewed in [12]).

The central tenet of studying changes in DNA methylation is that it represents a major mechanism by which chromatin access of transcription factors (TF) and the basal transcriptional machinery is modulated. There are at least 50 years of research supporting links between altered DNA methylation of genes that, in turn, govern processes associated with cancer initiation and progression. In the 1960s, researchers had already observed altered patterns of DNA methylation in cancer cell models and even proposed that this resulted in altered distribution of the sites of transcription initiation [13,14]. The biochemistry and regulation of CpG methylation was investigated and led to the description of altered methylation states of known tumor suppressors and oncogenes. Subsequent exploration of the associations at candidate sites between altered DNA methylation and gene expression largely confirmed the hypothesis that the DNA methylation signal serves as a physical impediment to TFs and the transcriptional machinery.

Thus, a general hypothesis emerged where the consequence of DNA methylation is to provide a physical barrier to positive regulators of transcription. More specifically, DNA methylation is a process of epigenetic control of the information encoded by DNA. That is, the process does not alter the underlying DNA sequence as 5-methylcytosine (5mC) still operates in a codon in the same manner as unmethylated cytosine, and yet it is heritable to daughter cells as the 5mC mark is copied to the nascent strand of DNA during DNA replication.

Methylated cytosine is a dynamic modification not only because it is added and removed by enzymatic processes, but also because it can spontaneously deaminate and, therefore, mutate [15,16,17]. By contrast, spontaneous deamination of unmethylated cytosine is readily recognized by the DNA repair machinery and corrected. Therefore, during the course of evolution methylated CpG dinucleotides have steadily been deaminated. As a result, across the human genome the frequency of CpG is actually underrepresented accounting for only 1/80th of the dinucleotides in the genome rather than the expected 1/16th [18].

The overwhelming majority (~85%) of methylated CpG sites in the genome are found in repetitive elements such as short interspersed nuclear elements (SINEs) and long interspersed nuclear elements (LINEs), as well as satellite DNA repeats in peri-centromeric regions. Persistent methylation of CpG in these repetitive elements is probably withstood as there is less evolutionary pressure on these regions and they can withstand the increased mutation rate without apparent harm to fitness. By contrast, CpG regions that remain unmethylated presumably have higher evolutionary pressure to remain unmethylated and thereby avoid the higher mutation rate. Approximately 15% of the CpG sites are found in CpG islands in the promoter regions of some 70% of protein-coding genes [19]. Presumably the regulatory function of methylation at CpG islands outweighs the potential mutation that may occur and argues for evolutionary conservation of CpG island function in gene regulation. Hence, while there is significant evolutionary pressure to reduce CpGs, the function of CpGs in islands is advantageous and protected from evolutionary erosion [20,21].

CpG islands are between 300 and 3000 bp (base pairs) in length with a GC content of greater than 50% and an observed/expected ratio of CpG to GpC greater than 0.6. Consistent with the idea that they play roles in the control of gene expression, they are often found to overlap with the histone mark trimethylated at Lysine 4 of Histone 3 (H3K4me3) and binding sites for RNA polymerase indicative of active, or at least permissive, transcription [22,23]. Although annotation of the non-coding genome is not as complete, there is evidence that CpG islands that previously appeared to be orphans, not associated with a known gene, can be associated with long non-coding RNA (lncRNA), micro RNAs (miRNAs) and other non-coding genes [24] and that these distal, or orphan, CpG islands may be important sites for TF binding and the control of non-coding RNA expression [25].

## 2. The Control of CpG Methylation and Its Impact on Transcription

As with all modifications to DNA, the addition and subtraction of methyl groups is tightly regulated by antagonistic enzyme families. The DNA methyl transferases (DNMTs) add methyl groups. DNMT1 is the major DNA methyltransferase expressed at high levels in all tissues where it plays a role in the maintenance of cytosine methylation following progression though the cell cycle. DNMTA3a and 3b are more involved in the de novo initiation of methylation patterns. Whilst these enzymes have been identified and investigated for many years, being cloned in the late 1980s and early 1990s [26], the ten-eleven translocation (TET) family of proteins were identified as the methylcytosine dioxygenases only in 2009 [27]. These proteins can reverse the methylation actions of DNMTs by oxidizing 5mC. Again, underscoring the importance of DNA methylation in embryogenesis, the genetic knockout of DNMT and TET family members display a range of embryonic phenotypes, including lethality, supporting the importance of normal regulation of DNA methylation [28,29,30,31,32,33,34,35,36].

DNMT1 maintains the DNA methylation pattern from the parent cell to the daughter cell. This heritable nature of DNA methylation is a key feature defining DNA methylation as an epigenetic mark. Unlike DNMT1, DNMT3a and DNMT3b normally methylate DNA that is unmethylated on both strands and do not have binding preference to the hemi-methylated state—a feature central to DNMT1’s maintenance function. The roles of DNMT3b and 3a are not completely redundant. DNMT3a is a distributive enzyme, while DNMT3b is a processive enzyme. DNMT3a is important for focal methylation of single copy genes or regions where there are not long stretches of CpG to methylate. On the other hand, the high processivity of DNMT3b is conducive to its role in methylating the highly repetitive peri-centromeric regions where there are long stretches with many CpG positions to be methylated. Another DNMT3 family member is DNMT3L, which has no catalytic activity because of having a non-consensus catalytic domain. Nevertheless, DNMT3L plays an important role in DNA methylation because it interacts with DNMT3a and 3b. For instance, the interaction with DNMT3a increases the activity of DNMT3a and has been shown to be essential for maternal imprinting.

TET1 was initially cloned from leukemic cells where it was identified as a fusion partner of the mixed-lineage leukemia (MLL) translocation [37], but its function was only revealed by in 2009 [27], when the capacity to oxidize 5mC was revealed. Three TET family members exist [38], and similarly to the DNMTs, the TETs appear to differ in their functions. TET1 exerts an important embryonic function by governing expression of stem cell specific TFs such as NANOG [38] and its loss triggers a shift towards trophectoderm differentiation. TET1 also governs meiosis [39] and prevent the spread of CpG methylation [40]. Others have applied TET1 chromatin immunoprecipitation combined with next generation sequencing (ChIP-Seq) to approaches revealed binding of TET1 to CpG rich sequences in transcriptionally-active promoters, but also at polycomb-repressed genes. Thus, TET1 at least has a potentially dual role in triggering gene activation, and also modulating polycomb-mediated gene repression [41,42]. Again, in embryonic systems, TET1 was shown to associate with so-called bivalent environments with H3K4me3 and H3K27me3, adding evidence to the concept that TET1 at least controls the so-called ‘poised’ chromatin state for example at developmentally-regulated genes [43]. Supporting an antagonistic role against DNMTs, TET1 can remove the imprinted status of specific genes [44,45].

There is some evidence for TET2 and TET3 to target specific gene regulation programs, for example the active repression of interleukin 6 (IL6) during inflammation through active recruitment of histone deacetylases (HDAC) independently of modification of 5mC [46]. There is also evidence of either genetic variation or somatic mutation of TET2 to impact hematopoietic stem cell function and is implicated in a range of myeloid disorders [47].

Thus, 5mC is directly governed spatially and temporally, often in a gene program specific manner, by at least six different enzymes. The levels of 5mC at CpG regions and islands is in turn sensed by a number of proteins, to help translate the chemical signal of the methyl group into biological functions that include regulating transcriptional activity; this is most clearly found when the CpG island is in a proximal promoter region.

The impact of altered CpG island methylation is thought to regulate transcription in at least two mechanisms. Firstly, the increase in methylation levels at CpG islands or CpG regions can impact the physical access of TFs and, therefore, suppress gene regulation. Indeed, early studies demonstrated that a single methylated CpG in a 6 bp region could impact TF access [48]. Secondly, the methylation of CpG regions are, in turn, recognized by a family of proteins containing the methyl-CpG-binding-domain, known collectively as MBDs. These proteins Methyl-CpG Binding Protein 2 (MeCP2), Methyl-CpG Binding Domain Protein 1 (MBD1), MBD2, MBD3, and MBD4), along with Zinc Finger and BTB Domain Containing 33 (ZBTB33/KAISO) which has a different domain to recognize methylated DNA, are found in complexes that contain other chromatin modifying enzymes such as HDACs. Various workers established that CpG methylation attracts MeCP1 [49] and that these proteins can recruit HDACs to repress transcription [50]. Similarly, MeCP2 binds to methylated DNA and recruits SIN3a, which recruits HDACs leading to a situation where regions of DNA methylation coexist with regions of deacetylated histones that can form a compact, closed chromatin structure to exclude interaction with TF and the basal transcriptional machinery. Therefore, the levels of methylation at CpG islands can impact more widely the genome around the island.

When restricted to specific biological settings, the relationships between DNA methylation, chromatin assembly and transcription are certainly apparent. The mostly-methylated CpG islands on the inactive X chromosome in a female cell strongly (but not exclusively) correlate with gene silencing [51,52,53]. In a similar way, imprinted genes, expressed either from the paternal or the maternal allele, are associated with CpG island regions methylated only on one allele. Another group of genes, the cancer-testis (CT) antigen genes, such as those of the melanoma antigen family (MAGE) families often have methylated CpG island promoters in all normal tissues, except testes, where they are expressed. Often these genes are expressed widely in cancers where those CpG islands lose methylation [54,55,56,57,58,59].

## 3. The Transition from Epigenetic to Epigenomic Analyses of Cancer Genomes

### 3.1. Development of Technologies and Computational Approaches for Epigenomic Analyses

Early work in cancer systems, at candidate loci, quickly revealed that gains and losses of DNA CpG methylation were significantly detected at the sites of tumor suppressors and oncogenes. These observations gave rise to the concept of epi-mutations that could act alongside somatic mutations as cancer-drivers [60]. Specifically, regulatory and promoter CpG regions on these genes have been identified as being inappropriately methylated. Some of the earliest studied in the field were focused on loss of methylation at the CpG islands of known oncogenes, for example at the RAS locus [61]. Subsequently, other workers considered the possibility for gain of methylation at tumor suppressors and again early studies demonstrated gain of CpG methylation at the calcitonin gene in lung cancer [62]. In part, this suggested that hypomethylation at oncogenes may arise earlier in cancer, or pre-malignant conditions whereas hypermethylation, by contrast, possibly arises later and tends to be associated with promoters that control the expression of oncogenes and tumor suppressor. More recently, there is some evidence for coordinated hypo and hypermethylation events occurring in leukemia [63].

As well as giving rise to the concept of epi-mutations, the sequencing of DNA methylation events, inter-joined as they are with the regulation of histone modifications, also justified development of DNMT inhibitors and the combination of epigenetic therapies that targeted both CpG methylation and repressive histone modifications [64,65].

These candidate gene studies were catalysts for the development of genomic technologies and large scale data-analytic approaches aimed at revealing how many and how frequently CpG islands and regions were methylated in various cancer types. For example, some of the first technologies to expand from the candidate loci to genome-wide coverage built on the use of methylation sensitive restriction enzymes that digest specifically unmethylated CpG regions [66]. The recovered fragments were modified to detect digestion, or not, of fragments indicative of methylation status that could be imaged with either radiolabel approaches or subsequently with next generation sequencing (NGS) approaches. Thus, workers were able to begin to measure quantitative differences in the levels and distribution of CpG methylation between cell models, or tumor material compared with adjacent normal material.

In parallel, other technologies were developed using microarray platforms and hybridization, and the Illumina platform clearly emerged as the market leader. This platform allowed the relatively easy scanning of multiple CpG regions in cells, be they cell lines, frozen tumor or even formalin fixed material. The CpGs assessed by these technologies were selected based on their relative position within CpG islands and or near to gene features such as Reference Sequence (RefSeq) annotated transcriptional start sites (TSS). Although these early arrays and more recent larger ones sample CpG positions in all known TSSs and CpG islands and even annotated enhancers, they are not exhaustive in their coverage and frequently have rather limited numbers of CpG positions representing large genomic regions. Furthermore, their design rationale is based on an incomplete understanding of which CpG positions might be most relevant to regulation of chromatin and of gene expression. Nonetheless, these approaches have very clearly established that there are altered patterns of CpG methylation at CpG islands in a wide range of cells and in health and disease.

A significant caveat is an implicit assumption that the CpG positions represented on the array from a given genomic region are indicative of the broader methylation state of all CpGs in that region. While this is clearly correct in some situations, it may not be universal, and there is clearly emerging evidence for highly specific patterns of CpG methylation. For example, when comparing CpG island methylation between normal and cancer, it is likely that a few CpG positions can accurately represent the CpG island status. However, it is much less clear if this is true when comparing non-malignant genomes with different exposures. Nevertheless, array studies have provided significant insights from which a generally more textured understanding has emerged of the roles of different CpG methylation positions and density on gene expression in development (reviewed in [67,68]). Indeed, the CpG methylation arrays are one of the earliest and widely applied genomic technologies, and also helped to catalyze the development of the statistical framework for the analyses of differential DNA methylation [69,70].

To obtain exhaustive genome-wide coverage has required the development of NGS-based approaches to DNA methylation [71,72], for example, whole genome bisulphite sequencing (BS) at the base pair resolution. However, this does not distinguish between 5mC and 5-hydroxymethylcytosine (5hmC) and, therefore, comparative analyses is needed using BS and oxidative BS sequencing. 5mC and 5hmC specific antibodies can be used in Methylated DNA immunoprecipitation (MeDIP-Seq) approaches. Other modifications include GC based enriched regions (eRRBS), as well as locus-averaging methods that pull down methylated peaks or associated with MBD binding. Array technology has continued to be developed, principally by Illumina who have developed 27K, 450K and no 850K Infinium Methylation EPIC rrays for more comprehensive analyses.

Marching in tandem with the progress of the technologies available to survey the CpG status across the genome have been an equally profound development of the computational approaches to analyze the data. The broad demands of these software are to cope with modelling the data, dealing with incompleteness of data and identifying differentially methylated regions (DMR). Most commonly, the bioinformatics community uses the R platform for statistical computing [73,74] and a range of library packages implemented in Bioconductor [75,76]. For example, there are currently over 60 packages available in Biconductor that deal with the analyses of genomic CpG methylation data. R and Bioconductor are both community developed and maintained and, therefore, new approaches are continually developed. Indeed, Bioconductor illustrates the combination of packages, or software libraries, that can be applied for optimal workflows for many common bioinformatic analyses. Such a workflow has been developed for the analyses of DNA methylation [77].

### 3.2. Analyses of CpG Methylation in Large Cohorts of Publically Available Data

In tandem with the development of these, and other, genomic technologies has been their widespread application across multiple genomes undertaken by researchers in the Encyclopedia of DNA elements (ENCODE) [78,79], RoadMap Epigenome [80], and FANTOM [81] consortia. In doing so, these consortia have also generated remarkable volumes of publically available data with which to interrogate DNA methylation patterns and relationships to gene expression and cell phenotypes. In cancer it is clear that the TF-genome interactions are corrupted [82,83,84,85] and “re-wired” [86,87,88], for example, by somatic mutations and endogenous structural variants that disrupt TF binding. A major driver of addressing global DNA methylation in cancer has been the development of large cancer genome studies, for example the Cancer Genome Atlas (TCGA) in which virtually all 30,000 tumors in the archive have been screened with Illumina microarray approaches. This, in part, has allowed workers to undertake pan-cancer analyses of the DNA methylation patterns in an effort to classify different methylation subgroups between tumors [89].

However, testing the extent of genome-wide correlations between CpG methylation and gene expression is challenging because of statistical, biological, and technical limitations and incomplete biological understanding and, therefore, the extent and strength of correlations differ significantly between studies. For example, it is also critical to consider that these DNA methylation states are in the context of chromatin, and that this chromatin structure is, in part, being defined by modifications of histones making up the nucleosomes; unmethylated CpGs are frequently associated with active histone marks. This interplay of epigenetic events has also guided how researchers consider the transcriptional potential of a gene, as to whether the gene is active, repressed, or poised [90]. If the gene locus was in a transcriptionally permissive environment, and is poised, then methylation can impact expression of the gene.

A further major impediment to identifying TF-genomic interactions that are disrupted by CpG methylation, and which might also drive cancer development is the sheer volume of TF complexes involved and the combinatorial nature of epigenomic events. Over 20% of the protein-coding genome relates to transcriptional control; approximately 2500 TF interact with over 2000 TF co-factors and chromatin remodeling factors, before even considering the actions of the non-coding genome. The diversity of TF-genomic interactions is amplified even further when considering that each TF complex may have thousands of genomic binding sites, known collectively as a cistrome [91].

Therefore, it is reasonable to postulate that the choice of TF binding sites is guided by the interplay of multiple histone modifications and the CpG methylation status, but it is much harder to test the strength of these relationships and how they diverge across cancer states. Therefore, considering the impact of CpG methylation on TF-genomic interactions quickly becomes a very challenging question. For instance, although CpG islands tend to be considered as discrete data points, being either on or off, they are in fact highly continuous. CpG status can be altered by both changes in the distribution and density of methylation.

Furthermore, workers have more recently proposed that the regions around the islands, (the shores and shelves) carry further important information to mediate relationships that control gene expression [92,93]. For example, the shores and shelves on CpG islands tend to have higher variation across cancers and therefore comparative analyses have to be specific to locations. As the CpG methylation status of the human genome has become increasingly mapped it has also emerged that differential methylation is not restricted to the CpG islands, but also extends to CpG regions for example at enhancers [94] as well as across gene bodies. Again, it is worth remembering that the one of the earliest studies of DNA methylation impacting transcription factor binding considered a single 6 bp region and the impact of one methylated cytosine within that region [48]. Thus, as further details regarding DNA methylation patterns in tissues have emerged, so too have the complexities in which they relate to transcription.

From a developmental perspective, recent genomic studies have begun to reveal the impact of CpG methylation at enhancer and intergenic regions. Clearly, 5mC dynamics are dramatic and dynamic in embryos to establish totipotency. In mammals, active TET-dependent oxidation of 5mC as well as passive cell-division dependent depletion is critical to demethylate gene enhancers and activation of transcription factors that control embryonic development pathways [95]. More recently, cell-based studies have also modeled the impact of CpG methylation in gene enhancers and revealed the interplay with the so-called bivalent status of enhancers and super-enhancers [96], again demonstrating the interplay between CpG methylation and histone modifications.

There are approximately 50,000 CpG islands in the human genome (depending on the specific definitions of GC content, density, and length) and the shore and shelf concept of course expands this number. Rarely is a region entirely methylated or not and therefore the calculation of the level of methylation is not trivial, requiring relatively sophisticated statistical models that aim to identify DMR. To ascribe function to these DMR requires some aspect of spatial annotation. For example, if a CpG island is proximal to a TSS it is a reasonable assumption that heavily methylated (or unmethylated) status impacts the expression of this gene. This does not preclude the fact that methylation may impact the expression of distal genes in both the 5′ and 3′ direction, and that the promoter of one gene may actually be a distal enhancer of another [97] and, therefore, give rise to so-called “ripple” expression of adjacent genes [98,99]. Additionally, gene expression may be impacted by the combined effect of 5′ and 3′ distal and proximal regulatory regions and may include the methylation of the gene body.

Finally, the definition of a DMR may also relate to cell type. For example, in a cancer genome it is not clear if the average methylation state of a limited number of CpG probes within a 1 kb CpG island accurately define the overall methylation state of that island. If they do, it is also unclear that would also be true in normal matched tissues.

These concerns notwithstanding, NGS technologies have been applied to an ever-greater extent with increasing genomic coverage and resolution of CpG methylation yielding new observations regarding DNA methylation patterns. Genome-wide, in normal cells, there is a negative correlation for approximately 20% of the genome between elevated 5mC in CpG islands and repression of a neighboring TSS [100].

Thus, with the development and widespread application of tools to measure CpG methylation levels and distribution across the genome it is now possible to test the extent of correlation between CpG methylation and gene expression. Within the cancer context the remarkable volume of data developed by TCGA investigators has allowed investigators to dissect the relationships between CpG methylation and gene expression.

One of the earlier studies by Aran et al. [101] addressed this question and focused on negative correlations between CpG methylation at enhancer sites and the gene body, and gene expression across multiple cell lines, and offered evidence that enhancer methylation was selectively disrupted in cancer. However, if the starting point are the individual datasets, rather than modelling the ones where there is the strongest negative correlation, then identifying these relationships de novo can be more challenging.

Combining MeDIP-Seq and ribonucleic acid (RNA)-Seq datasets from malignant mesothelioma cells was undertaken to investigate the genome-wide extent of strong negative correlations between methylation and gene expression. The number of such CpG methylation-gene expression negative correlations were strikingly small. For example, there were several thousand hypermethylated and hypomethylated genes but only hundreds of genes differential expressed, with the clearest relationships being at intronic regions where altered methylation associated with altered expression [102].

Similarly in breast cancer, genome-wide NGS approaches have been applied to identify how CpG methylation impacts gene expression and the emergence of drug resistance. In this context, approximately one thousand genes had both altered methylation and altered expression, perhaps supporting the concept that epigenetic events can allow for the rapid adaptation to drug exposure. However, drilling into the subset of data where CpG methylation and gene expression were negatively correlated identified fewer genes (in the low hundreds) [103]. In another report [104] in lung cancer the correlation between DNA methylation and gene expression was detected for approximately 750 genes, but for one third of these the correlation was positive. Again, a negative correlation could only be found for approximately 500 genes. Similarly, in esophageal cancer the authors only illustrated the negative correlation at candidate loci despite having generated RNA-Seq and matched MeDIP-Seq data [105].

Reflecting the challenges in finding strong and widespread negative correlations between genome wide CpG methylation and genes expression, investigators have applied more sophisticated analytical approaches to transform the continuous DNA methylation data into a categorical format. Again, in breast cancer, both positive and negative correlations between DMR and gene expression are often observed. By selecting for lowly-expressed genes enhanced the detection of greater negative correlations between gene promoter CpG methylation in the promoter [106]. Therefore, it seems that a negative correlation between CpG methylation and gene expression exists for only a small subset of genes expressed in cancer cells, numbering perhaps in the hundreds.

## 4. Prostate Cancer as a Model of the Interplay between Genomic and Epigenomic Cancer Drivers

Amongst men in the US, prostate cancer (PCa) is most common non-cutaneous cancer diagnosed and second leading cause of death [107,108]. This cancer is highly heterogeneous in terms of progression rates. Although pathological tumor grade (Gleason Grade) accurately predicts disease outcome, currently clinical parameters that can be exploited before surgery do not accurately predict progression risks to more aggressive stages of disease. Therefore, it is not easy to identify men who both need and will be cured by surgical treatment, from those men who will experience subsequent treatment failure and disease recurrence [109,110]. This is of clinical significance as patients who experience treatment failure are significantly more likely to progress to more aggressive forms of PCa with significantly increased risks of tumor-related mortality [111,112].

This ambiguity is further obscured because the incidence and natural history of PCa varies between races. American men of African ancestry have a 19% increased incidence, and 37% increased mortality from PCa compared to men of European ancestry (reviewed in [113,114,115,116]). Thus, in African American (AA) PCa patients, the disease appears more aggressive, and occurs at a younger age, than European American (EA) patients.

In an effort to more accurately define disease multiple groups [117], including the TCGA consortium, have added to previous understanding [118,119] and established roles for common genetic alterations in PCa [120,121,122], and novel somatic mutations, including Forkhead Box A1 (FOXA1), Speckle-Type POZ Protein (SPOP). Also supporting the importance of androgen receptor (AR) signaling in PCa and the cross-talk with epigenetic events, the co-activator Nuclear Receptor Coactivator 2 (NCOA2) is commonly amplified [122,123].

The complex nature of cancer phenotypes however cannot be explained by genetic components alone [124]. Epigenomic modifications and events contribute significantly to cell transformation and play distinct yet complementary roles to genomic events, and add to a fuller explanation for the etiology of disease. For example, up-regulation of the histone methyltransferase, Enhancer of Zeste Homolog 2 (EZH2) appears common in both localized and metastatic PCa, and associates with poorer prognosis [125,126]. Additionally, reflecting different genomic and epigenomic drivers of PCa, there are significant global differences in the pattern of CpG DNA methylation associated with different genetic PCa phenotypes, notably in the presence of *TMPRSS2–ERG* translocations [127,128]. Similarly, we and others have examined the expression and CpG methylation status associated with miRNA (reviewed in [129]). For example, in PCa, promoter hypermethylation is associated with loss of microRNA (miR)-200 family members that regulate cell migration/invasion. We have defined cohorts of miRNA that predict aggressive disease [130] and in turn revealed that their expression may often be associated with altered CpG methylation [131].

The changes in CpG methylation in PCa progression have been comprehensively reviewed by Lynch and co-workers [132]. They highlighted the consistency of methylation at the promoter of certain genes, for example Glutathione *S*-Transferase Pi 1 (GSTP1). They also combined datasets and measured the overlaps to identify expression of 168 genes commonly identified to have associated DMR and altered expression including *GSTP1* and others such as retinoic acid receptor β (*RARB*), Ras Association Domain Family Member 1 (*RASSF1*), and Aldehyde Oxidase 1 (*AOX1*) as well homeobox gene family members.

Others have sought to relate CpG methylation patterns to clinical outcome and combined their patterns in univariate regression analyses of time to disease recurrence and revealed that methylation of certain loci, for example again including *AOX1* and *RARB* [133] predicted disease progression [134,135]. Further supporting the relevance of DMR in PCa progression, the TCGA investigators revealed how altered DNA methylation patterns associated with different PCa genetic phenotypes. Interestingly, no negative correlation patterns were noted for DNA methylation level and either mRNA or miRNA expression. However, subsequent studies by Jin and co-workers of the same data modelled the interplay between distal, promoter, and genic CpG methylation and gene expression [136]. Notably, these workers revealed that TSS and distal hypermethylation, but not hypomethylation, were associated with differential gene expression. Again, reflecting other cancer studies, the correlations were both negative and positive and the number of genes for which a specific location of hypermethylation negatively correlated with gene expression was fewer than 100.

To complement these studies, many researchers have examined how the expression and genetic variation of either DNMTs or TETs are altered in cancer systems. For example, earlier studies have linked gain of expression or genetic variation with altered DNA methylation patterns in various tumors. In many cases these gains of DNMTs function were linked to disease progression and worse clinical features. Indeed, the interplay of DNMTs and TETs has also been established as a putative feed forward loop where increased DNMTs function silences TETs [137]. Furthermore, researchers have examined how DNMTs and TETs may contribute to disease progression and established roles for increased DNMTs in mouse models of prostate cancer [138,139,140] and that TET1 for example is disrupted by copy number changes correlating with reduced 5hMC levels in prostate cancer samples [141]. Others have revealed a targeted function for TET1 to interact with the pioneer factor FOXA1 to activate lineage-specific enhancers [142].

A complementary area of very active research in PCa is the control of one-carbon metabolism and the methionine cycle that generates the SAM pools that in turn feed into the control of DNA methylation, as well as histone methylation [143]. This pathway has unique relevance to the prostate which normally secretes high levels of acetylated polyamines which in turn can stress the cellular production of SAM and, therefore, the biochemistry of prostate epithelial cells is modified to enhance methionine salvage pathways. Indeed this dependency may highlight a unique therapeutic approach to targeting prostate cancer through inhibitors of Methylthioadenosine Phosphorylase (MTAP), the rate limiting enzyme [144]. Given that folate is an upstream dietary-derived precursor of these pathways it is likely that dissecting the links between folate metabolism and prostate cancer may yield unique and tissue-specific insight [145,146,147].

## 5. Bioinformatic Approaches to Reveal Associations between DNA Methylation and Prostate Tumor Status

To add to these studies, we have now sought to model the impact of the DNA methylation pathway on gene expression in PCa by using the R platform for statistical computing and a range of library packages implemented in Bioconductor. As a starting point, we created a list of genes known to be involved in the control of DNA methylation. To do this we downloaded genes from the DNA methylation pathway from WikiPathways [148] and combined these genes with those returned from searches of DNA methylation pathway genes in UniProt [149]. Together these approaches yielded a unique list of 165 genes includes those that control the regulation of SAM pools and DNA CpG methylation.

**Figure 1:**
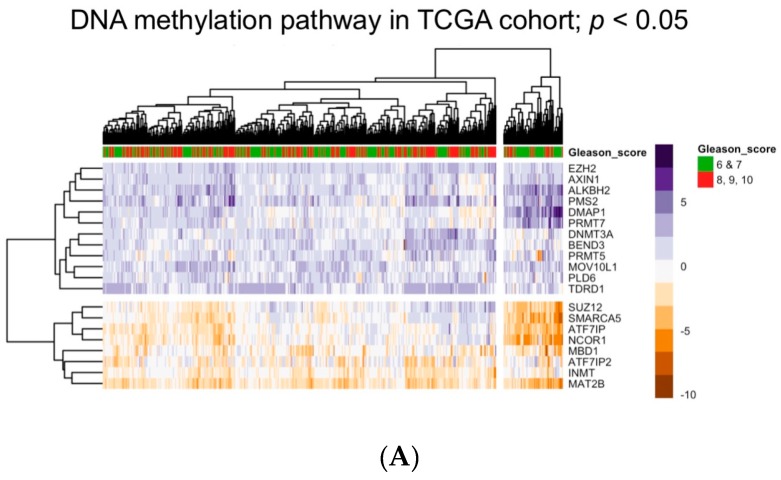

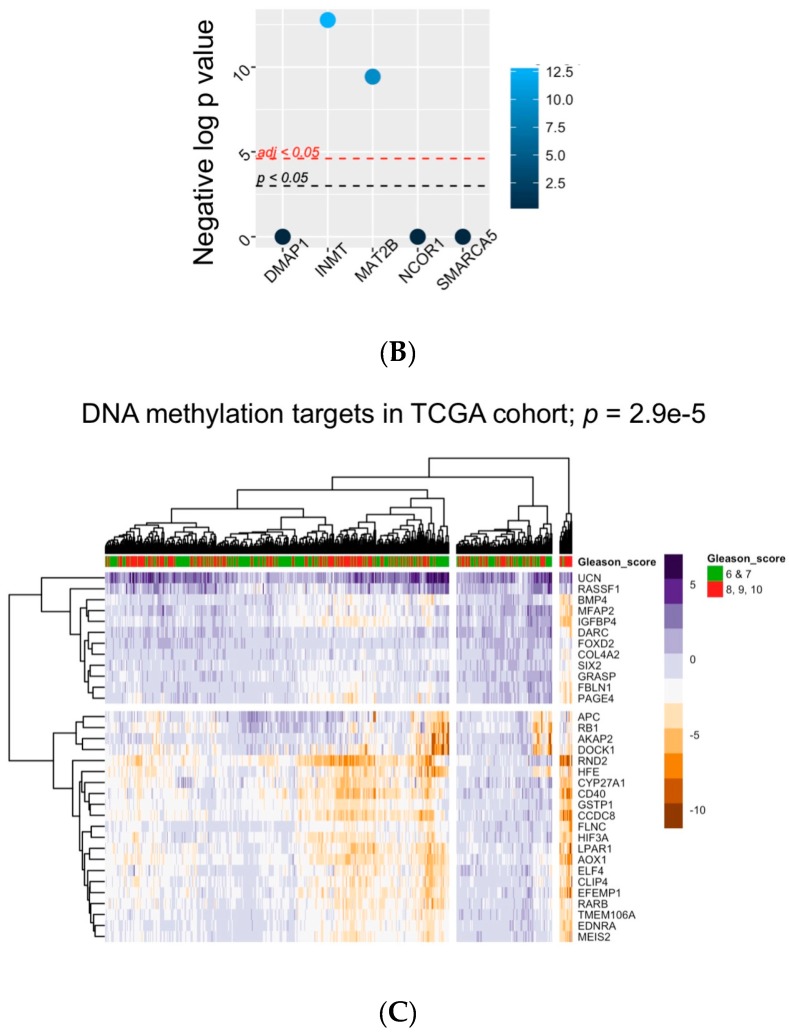
Expression and association of the DNA methylation pathway genes with tumor outcome. (**A**) Heatmap illustrating common and significantly altered mRNA expression of the DNA methylation pathway in the Cancer Genome Atlas (TCGA) prostate cancer (PRAD) cohort (*n* = 497). Gene expression was measured as normal tissue relative *Z*-scores of all genes detectable in at least 80% of tumors. Cluster membership significantly identifies aggressive tumors (*p* < 0.006); (**B**) The negative and positive correlation (Pearson correlation either <−0.6 or >0.6) for each of these commonly altered genes from the DNA methylation pathway (*n* = 21) and all other detectable genes in the TCGA cohort (*n* = 16,785) was measured and the enrichment of the 165 common targets of DNA methylation from the Lynch et al. review [132] was measured using a hypergeometric test. Only the indicated genes had significant correlation with all genes and significant enrichment of the targets of DNA methylation; (**C**) Heatmap illustrating common and significantly altered mRNA expression of genes that significantly correlate with Indolethylamine *N*-Methyltransferase (INMT) and Methionine Adenosyltransferase 2B (MAT2B) and are known targets of DNA methylation in TCGA PRAD cohort. Cluster membership significantly identifies aggressive tumors (*p* < 0.0007).

To examine how these genes were altered in PCa we examined their expression in the TCGA prostate cancer cohort (PRAD) of 497 tumors. These data are publically available and were downloaded. The data actually include tumors and normal samples and, therefore, we created an expression table of all genes detectable in at least 80% of tumors (*n* = 16,785) given as relative *Z*-scores as compared to the mean of the normal [150]. Expression of the 165 gene-panel of the DNA methylation pathway was examined in this table using genefilter to capture only those genes altered by more than two *Z*-scores in 25% of tumors; this yielded 21 genes on the DNA methylation pathway. These genes included DNA methyltransferase (*DNMT3A*), *MBD1*, and Nuclear receptor corepressor (*NCOR*)*1*. Tumor expression patterns for these genes were then visualized and clustered by expression on a heatmap (pheatmap). Relationships between cluster membership and tumor grade (Gleason Grade 6 and 7 compared to 8, 9, 10) were measured using survival. The expression patterns of these 21 genes clustered tumors into groups that in turn associated with significantly different levels of Gleason Grade (*p* < 0.006) (Figure 1A). Interestingly, of these genes shown to be associated with altered CpG methylation, we had previously identified that increased NCOR1 binding to gene targets in prostate cancer cell lines leads to elevated CpG methylation [151].

Next, we sought to investigate the relationships between these 21 genes and the targets of DNA methylation. To do this, we identified all genes amongst the 16,785 genes expressed in the TCGA-PRAD tumors that positively and negatively correlated with each of these 21 genes on the DNA methylation pathway. Subsequently we used a hypergeometric test to measure how genes that were strongly correlated with these 21 genes on the DNA methylation pathway were themselves enriched for genes that were known targets of DNA methylation. Thus, for each of the 21 DNA methylation pathway genes the negative and positive correlations (Pearson correlation either <−0.6 or >0.6) of expression was determined. Only five DNA methylation pathway genes had strong correlations and subsequently, the enrichment of the 165 common targets of DNA methylation from the Lynch et al. review [132] was measured within these correlated genes, using a hypergeometric test.

This analysis identified that from the DNA methylation pathway genes only Indolethylamine *N*-Methyltransferase (INMT) and Methionine Adenosyltransferase 2B (MAT2B significantly correlated with a set of genes which themselves were significantly enriched for known targets of DNA methylation in PCa (Figure 1B). For example, INMT and MAT2B were commonly altered and associated with the expression of genes that are significantly enriched for known targets of DNA methylation changes in PCa. INMT is a methyltransferase and methionine adenosyltransferase. MAT2B regulates the biosynthesis of SAM from methionine and therefore is important in the regulation of SAM pools upstream of DNA methylation events. INMT was previously identified as predicting disease progression risk in prostate cancer [152] whereas MAT2B has not previously been implicated in prostate cancer risk or etiology. Together, these finding suggest that mechanisms that control the flux of SAM pools appear to be linked to aggressive PCa and that only a relatively small subset of genes are probably targeted. Again, it is worth emphasizing that SAM pools are related to diet and genetic variation, and there is a significant literature over how control of the central methyl donor impacts a range of diseases including PCa [144,145,153,154,155,156,157,158].

Next, we took targets of DNA methylation that correlated with INMT and MATB expression and identified those with the most altered expression in the tumors with higher Gleason Grade tumors; this was 32 genes (Figure 1C). These targets included of course known targets of DNA methylation and included genes that were both up and down-regulated. Within the down-regulated genes (*n* = 20) the down-regulated expression was pronounced and significant in the more aggressive; these genes included RARB, AOX1, and GSTP1.

Together this translational bioinformatics approach has mined existing datasets and identified that 21 members of the DNA methylation pathway are commonly altered and associated with more aggressive tumor features (e.g., INMT and MAT2B). These genes were strongly correlated with a small number of known targets of DNA methylation including RARB, which in turn could distinguish tumors with higher Gleason Grade.

## 6. Summary

The current review has aimed to examine the central aspects of the relationships between DNA CpG methylation and the control of gene expression. Within the cancer arena, this complex field has made very significant strides with the application of genome-wide technologies and sophisticated statistical approaches, combined with high-quality and large-scale tumor profiling data. Perhaps surprisingly, the numbers of negative correlations between DNA CpG methylation and gene expression are in the hundreds not thousands, although within these there are clear examples of tumor adaptation to drug exposure, and genes that are known tumor-drivers. We also present a bioinformatics pipeline to examine how genes known to control DNA CpG methylation relate to altered gene expression and tumor status. This approach is relatively generic and revealed that, at least in PCa, the control of the biosynthesis of SAM is significantly associated with altered gene expression and tumor aggressiveness.

## References

[1] Bird A. (2002). DNA methylation patterns and epigenetic memory. Genes Dev..

[2] Lister R., Mukamel E.A. (2015). Turning over DNA methylation in the mind. Front. Neurosci..

[3] Jones M.J., Goodman S.J., Kobor M.S. (2015). DNA methylation and healthy human aging. Aging Cell.

[4] Niculescu M.D., Lupu D.S. (2011). Nutritional influence on epigenetics and effects on longevity. Curr. Opin. Clin. Nutr. Metab. Care.

[5] Esteller M. (2007). Cancer epigenomics: DNA methylomes and histone-modification maps. Nat. Rev. Genet..

[6] Kumar S., Cheng X., Klimasauskas S., Mi S., Posfai J., Roberts R.J., Wilson G.G. (1994). The DNA (cytosine-5) methyltransferases. Nucleic Acids Res..

[7] Matzke M.A., Mosher R.A. (2014). RNA-directed DNA methylation: An epigenetic pathway of increasing complexity. Nat. Rev. Genet..

[8] Kouzarides T. (2002). Histone methylation in transcriptional control. Curr. Opin. Genet. Dev..

[9] Guenther M.G., Jenner R.G., Chevalier B., Nakamura T., Croce C.M., Canaani E., Young R.A. (2005). Global and Hox-specific roles for the MLL1 methyltransferase. Proc. Natl. Acad. Sci. USA.

[10] Mentch S.J., Locasale J.W. (2016). One-carbon metabolism and epigenetics: Understanding the specificity. Ann. N. Y. Acad. Sci..

[11] Mattocks D.A., Mentch S.J., Shneyder J., Ables G.P., Sun D., Richie J.P., Locasale J.W., Nichenametla S.N. (2016). Short term methionine restriction increases hepatic global DNA methylation in adult but not young male C57BL/6J mice. Exp. Gerontol..

[12] Nazki F.H., Sameer A.S., Ganaie B.A. (2014). Folate: Metabolism, genes, polymorphisms and the associated diseases. Gene.

[13] Scarano E., Geraci G., Rossi M. (1967). Deoxycytidylate aminohydrolase. II. Kinetic properties. The activatory effect of deoxycytidine triphosphate and the inhibitory effect of deoxythymidine triphosphate. Biochemistry.

[14] Geraci G., Rossi M., Scarano E. (1967). Deoxycytidylate aminohydrolase. I. Preparation and properties of the homogeneous enzyme. Biochemistry.

[15] He X., Tillo D., Vierstra J., Syed K.S., Deng C., Ray G.J., Stamatoyannopoulos J., FitzGerald P.C., Vinson C. (2015). Methylated Cytosines Mutate to Transcription Factor Binding Sites that Drive Tetrapod Evolution. Genome Biol. Evol..

[16] Stier I., Kiss A. (2013). Cytosine-to-Uracil Deamination by SssI DNA Methyltransferase. PLoS ONE.

[17] Cannistraro V.J., Taylor J.S. (2009). Acceleration of 5-methylcytosine deamination in cyclobutane dimers by G and its implications for UV-induced C-to-T mutation hotspots. J. Mol. Biol..

[18] Hermann A., Gowher H., Jeltsch A. (2004). Biochemistry and biology of mammalian DNA methyltransferases. Cell. Mol. Life Sci..

[19] Sandelin A., Carninci P., Lenhard B., Ponjavic J., Hayashizaki Y., Hume D.A. (2007). Mammalian RNA polymerase II core promoters: Insights from genome-wide studies. Nat. Rev. Genet..

[20] Zemach A., McDaniel I.E., Silva P., Zilberman D. (2010). Genome-wide evolutionary analysis of eukaryotic DNA methylation. Science.

[21] Doi A., Park I.H., Wen B., Murakami P., Aryee M.J., Irizarry R., Herb B., Ladd-Acosta C., Rho J., Loewer S. (2009). Differential methylation of tissue- and cancer-specific CpG island shores distinguishes human induced pluripotent stem cells, embryonic stem cells and fibroblasts. Nat. Genet..

[22] Guenther M.G., Levine S.S., Boyer L.A., Jaenisch R., Young R.A. (2007). A chromatin landmark and transcription initiation at most promoters in human cells. Cell.

[23] Mikkelsen T.S., Ku M., Jaffe D.B., Issac B., Lieberman E., Giannoukos G., Alvarez P., Brockman W., Kim T.K., Koche R.P. (2007). Genome-wide maps of chromatin state in pluripotent and lineage-committed cells. Nature.

[24] Guttman M., Amit I., Garber M., French C., Lin M.F., Feldser D., Huarte M., Zuk O., Carey B.W., Cassady J.P. (2009). Chromatin signature reveals over a thousand highly conserved large non-coding RNAs in mammals. Nature.

[25] Medvedeva Y.A., Fridman M.V., Oparina N.J., Malko D.B., Ermakova E.O., Kulakovskiy I.V., Heinzel A., Makeev V.J. (2010). Intergenic, gene terminal, and intragenic CpG islands in the human genome. BMC Genom..

[26] Jin B., Robertson K.D. (2013). DNA methyltransferases, DNA damage repair, and cancer. Adv. Exp. Med. Biol..

[27] Tahiliani M., Koh K.P., Shen Y., Pastor W.A., Bandukwala H., Brudno Y., Agarwal S., Iyer L.M., Liu D.R., Aravind L. (2009). Conversion of 5-methylcytosine to 5-hydroxymethylcytosine in mammalian DNA by MLL partner TET1. Science.

[28] Li Z., Dai H., Martos S.N., Xu B., Gao Y., Li T., Zhu G., Schones D.E., Wang Z. (2015). Distinct roles of DNMT1-dependent and DNMT1-independent methylation patterns in the genome of mouse embryonic stem cells. Genome Biol..

[29] Liao J., Karnik R., Gu H., Ziller M.J., Clement K., Tsankov A.M., Akopian V., Gifford C.A., Donaghey J., Galonska C. (2015). Targeted disruption of DNMT1, DNMT3A and DNMT3B in human embryonic stem cells. Nat. Genet..

[30] Egger G., Jeong S., Escobar S.G., Cortez C.C., Li T.W., Saito Y., Yoo C.B., Jones P.A., Liang G. (2006). Identification of DNMT1 (DNA methyltransferase 1) hypomorphs in somatic knockouts suggests an essential role for DNMT1 in cell survival. Proc. Natl. Acad. Sci. USA.

[31] Oka M., Meacham A.M., Hamazaki T., Rodic N., Chang L.J., Terada N. (2005). De novo DNA methyltransferases Dnmt3a and Dnmt3b primarily mediate the cytotoxic effect of 5-aza-2′-deoxycytidine. Oncogene.

[32] Hattori N., Abe T., Hattori N., Suzuki M., Matsuyama T., Yoshida S., Li E., Shiota K. (2004). Preference of DNA methyltransferases for CpG islands in mouse embryonic stem cells. Genome Res..

[33] An J., Gonzalez-Avalos E., Chawla A., Jeong M., Lopez-Moyado I.F., Li W., Goodell M.A., Chavez L., Ko M., Rao A. (2015). Acute loss of TET function results in aggressive myeloid cancer in mice. Nat. Commun..

[34] Lu F., Liu Y., Jiang L., Yamaguchi S., Zhang Y. (2014). Role of Tet proteins in enhancer activity and telomere elongation. Genes Dev..

[35] Dawlaty M.M., Breiling A., Le T., Barrasa M.I., Raddatz G., Gao Q., Powell B.E., Cheng A.W., Faull K.F., Lyko F. (2014). Loss of Tet enzymes compromises proper differentiation of embryonic stem cells. Dev. Cell.

[36] Tan L., Shi Y.G. (2012). Tet family proteins and 5-hydroxymethylcytosine in development and disease. Development.

[37] Ono R., Kumagai H., Nakajima H., Hishiya A., Taki T., Horikawa K., Takatsu K., Satoh T., Hayashi Y., Kitamura T. (2009). Mixed-lineage-leukemia (MLL) fusion protein collaborates with Ras to induce acute leukemia through aberrant Hox expression and Raf activation. Leukemia.

[38] Ito S., D’Alessio A.C., Taranova O.V., Hong K., Sowers L.C., Zhang Y. (2010). Role of Tet proteins in 5mC to 5hmC conversion, ES-cell self-renewal and inner cell mass specification. Nature.

[39] Yamaguchi S., Hong K., Liu R., Shen L., Inoue A., Diep D., Zhang K., Zhang Y. (2012). Tet1 controls meiosis by regulating meiotic gene expression. Nature.

[40] Jin C., Lu Y., Jelinek J., Liang S., Estecio M.R., Barton M.C., Issa J.P. (2014). TET1 is a maintenance DNA demethylase that prevents methylation spreading in differentiated cells. Nucleic Acids Res..

[41] Wu H., Zhang Y. (2011). Mechanisms and functions of Tet protein-mediated 5-methylcytosine oxidation. Genes Dev..

[42] Wu H., D’Alessio A.C., Ito S., Xia K., Wang Z., Cui K., Zhao K., Sun Y.E., Zhang Y. (2011). Dual functions of Tet1 in transcriptional regulation in mouse embryonic stem cells. Nature.

[43] Pastor W.A., Pape U.J., Huang Y., Henderson H.R., Lister R., Ko M., McLoughlin E.M., Brudno Y., Mahapatra S., Kapranov P. (2011). Genome-wide mapping of 5-hydroxymethylcytosine in embryonic stem cells. Nature.

[44] Shen L., Wu H., Diep D., Yamaguchi S., D’Alessio A.C., Fung H.L., Zhang K., Zhang Y. (2013). Genome-wide analysis reveals TET- and TDG-dependent 5-methylcytosine oxidation dynamics. Cell.

[45] Yamaguchi S., Shen L., Liu Y., Sendler D., Zhang Y. (2013). Role of Tet1 in erasure of genomic imprinting. Nature.

[46] Zhang Q., Zhao K., Shen Q., Han Y., Gu Y., Li X., Zhao D., Liu Y., Wang C., Zhang X. (2015). Tet2 is required to resolve inflammation by recruiting Hdac2 to specifically repress IL-6. Nature.

[47] Tefferi A., Abdel-Wahab O., Cervantes F., Crispino J.D., Finazzi G., Girodon F., Gisslinger H., Gotlib J., Kiladjian J.J., Levine R.L. (2011). Mutations with epigenetic effects in myeloproliferative neoplasms and recent progress in treatment: Proceedings from the 5th International Post-ASH Symposium. Blood Cancer J..

[48] Watt F., Molloy P.L. (1988). Cytosine methylation prevents binding to DNA of a HeLa cell transcription factor required for optimal expression of the adenovirus major late promoter. Genes Dev..

[49] Lewis J.D., Meehan R.R., Henzel W.J., Maurer-Fogy I., Jeppesen P., Klein F., Bird A. (1992). Purification, sequence, and cellular localization of a novel chromosomal protein that binds to methylated DNA. Cell.

[50] Jones P.A., Laird P.W. (1999). Cancer epigenetics comes of age. Nat. Genet..

[51] Makhlouf M., Ouimette J.F., Oldfield A., Navarro P., Neuillet D., Rougeulle C. (2014). A prominent and conserved role for YY1 in Xist transcriptional activation. Nat. Commun..

[52] Nora E.P., Lajoie B.R., Schulz E.G., Giorgetti L., Okamoto I., Servant N., Piolot T., van Berkum N.L., Meisig J., Sedat J. (2012). Spatial partitioning of the regulatory landscape of the X-inactivation centre. Nature.

[53] Umlauf D., Goto Y., Cao R., Cerqueira F., Wagschal A., Zhang Y., Feil R. (2004). Imprinting along the Kcnq1 domain on mouse chromosome 7 involves repressive histone methylation and recruitment of Polycomb group complexes. Nat. Genet..

[54] Zhang W., Barger C.J., Eng K.H., Klinkebiel D., Link P.A., Omilian A., Bshara W., Odunsi K., Karpf A.R. (2016). PRAME expression and promoter hypomethylation in epithelial ovarian cancer. Oncotarget.

[55] Liang P., Song F., Ghosh S., Morien E., Qin M., Mahmood S., Fujiwara K., Igarashi J., Nagase H., Held W.A. (2011). Genome-wide survey reveals dynamic widespread tissue-specific changes in DNA methylation during development. BMC Genom..

[56] Meklat F., Li Z., Wang Z., Zhang Y., Zhang J., Jewell A., Lim S.H. (2007). Cancer-testis antigens in haematological malignancies. Br. J. Haematol..

[57] Hong J.A., Kang Y., Abdullaev Z., Flanagan P.T., Pack S.D., Fischette M.R., Adnani M.T., Loukinov D.I., Vatolin S., Risinger J.I. (2005). Reciprocal binding of CTCF and BORIS to the NY-ESO-1 promoter coincides with derepression of this cancer-testis gene in lung cancer cells. Cancer Res..

[58] Honda T., Tamura G., Waki T., Kawata S., Terashima M., Nishizuka S., Motoyama T. (2004). Demethylation of MAGE promoters during gastric cancer progression. Br. J. Cancer.

[59] Sigalotti L., Coral S., Nardi G., Spessotto A., Cortini E., Cattarossi I., Colizzi F., Altomonte M., Maio M. (2002). Promoter methylation controls the expression of MAGE2, 3 and 4 genes in human cutaneous melanoma. J. Immunother..

[60] Sieber O.M., Tomlinson S.R., Tomlinson I.P. (2005). Tissue, cell and stage specificity of (epi)mutations in cancers. Nat. Rev. Cancer.

[61] Feinberg A.P., Vogelstein B. (1983). Hypomethylation distinguishes genes of some human cancers from their normal counterparts. Nature.

[62] Baylin S.B., Hoppener J.W., de Bustros A., Steenbergh P.H., Lips C.J., Nelkin B.D. (1986). DNA methylation patterns of the calcitonin gene in human lung cancers and lymphomas. Cancer Res..

[63] Kushwaha G., Dozmorov M., Wren J.D., Qiu J., Shi H., Xu D. (2016). Hypomethylation coordinates antagonistically with hypermethylation in cancer development: A case study of leukemia. Hum. Genom..

[64] Abdelfatah E., Kerner Z., Nanda N., Ahuja N. (2016). Epigenetic therapy in gastrointestinal cancer: The right combination. Therap. Adv. Gastroenterol..

[65] Griffiths E.A., Gore S.D. (2008). DNA methyltransferase and histone deacetylase inhibitors in the treatment of myelodysplastic syndromes. Semin. Hematol..

[66] Costello J.F., Fruhwald M.C., Smiraglia D.J., Rush L.J., Robertson G.P., Gao X., Wright F.A., Feramisco J.D., Peltomaki P., Lang J.C. (2000). Aberrant CpG-island methylation has non-random and tumour-type-specific patterns. Nat. Genet..

[67] Suzuki M.M., Bird A. (2008). DNA methylation landscapes: Provocative insights from epigenomics. Nat. Rev. Genet..

[68] Ma X., Wang Y.W., Zhang M.Q., Gazdar A.F. (2013). DNA methylation data analysis and its application to cancer research. Epigenomics.

[69] Kristensen V.N., Lingjaerde O.C., Russnes H.G., Vollan H.K., Frigessi A., Borresen-Dale A.L. (2014). Principles and methods of integrative genomic analyses in cancer. Nat. Rev. Cancer.

[70] Dedeurwaerder S., Defrance M., Bizet M., Calonne E., Bontempi G., Fuks F. (2014). A comprehensive overview of Infinium HumanMethylation450 data processing. Brief. Bioinform..

[71] Tang J., Fang F., Miller D.F., Pilrose J.M., Matei D., Huang T.H., Nephew K.P. (2015). Global DNA methylation profiling technologies and the ovarian cancer methylome. Methods Mol. Biol..

[72] Sarda S., Hannenhalli S. (2014). Next-generation sequencing and epigenomics research: A hammer in search of nails. Genom. Inf..

[73] Le Meur N., Gentleman R. (2012). Analyzing biological data using R: Methods for graphs and networks. Methods Mol. Biol..

[74] Dudoit S., Gentleman R.C., Quackenbush J. (2003). Open source software for the analysis of microarray data. Biotechniques.

[75] Gentleman R.C., Carey V.J., Bates D.M., Bolstad B., Dettling M., Dudoit S., Ellis B., Gautier L., Ge Y., Gentry J. (2004). Bioconductor: Open software development for computational biology and bioinformatics. Genome Biol..

[76] Huber W., Carey V.J., Gentleman R., Anders S., Carlson M., Carvalho B.S., Bravo H.C., Davis S., Gatto L., Girke T. (2015). Orchestrating high-throughput genomic analysis with Bioconductor. Nat. Methods.

[77] Maksimovic J., Phipson B., Oshlack A. (2016). A cross-package Bioconductor workflow for analysing methylation array data. F1000Reserch.

[78] Birney E. (2012). The making of ENCODE: Lessons for big-data projects. Nature.

[79] Consortium E.P., Birney E., Stamatoyannopoulos J.A., Dutta A., Guigo R., Gingeras T.R., Margulies E.H., Weng Z., Snyder M., Dermitzakis E.T. (2007). Identification and analysis of functional elements in 1% of the human genome by the ENCODE pilot project. Nature.

[80] Roadmap Epigenomics C., Kundaje A., Meuleman W., Ernst J., Bilenky M., Yen A., Heravi-Moussavi A., Kheradpour P., Zhang Z., Wang J. (2015). Integrative analysis of 111 reference human epigenomes. Nature.

[81] Sanli K., Karlsson F.H., Nookaew I., Nielsen J. (2013). FANTOM: Functional and taxonomic analysis of metagenomes. BMC Bioinform..

[82] Weinhold N., Jacobsen A., Schultz N., Sander C., Lee W. (2014). Genome-wide analysis of noncoding regulatory mutations in cancer. Nat. Genet..

[83] Ongen H., Andersen C.L., Bramsen J.B., Oster B., Rasmussen M.H., Ferreira P.G., Sandoval J., Vidal E., Whiffin N., Planchon A. (2014). Putative cis-regulatory drivers in colorectal cancer. Nature.

[84] Bailey S.D., Desai K., Kron K.J., Mazrooei P., Sinnott-Armstrong N.A., Treloar A.E., Dowar M., Thu K.L., Cescon D.W., Silvester J. (2016). Noncoding somatic and inherited single-nucleotide variants converge to promote ESR1 expression in breast cancer. Nat. Genet..

[85] Oldridge D.A., Wood A.C., Weichert-Leahey N., Crimmins I., Sussman R., Winter C., McDaniel L.D., Diamond M., Hart L.S., Zhu S. (2015). Genetic predisposition to neuroblastoma mediated by a LMO1 super-enhancer polymorphism. Nature.

[86] Chng K.R., Chang C.W., Tan S.K., Yang C., Hong S.Z., Sng N.Y., Cheung E. (2012). A transcriptional repressor co-regulatory network governing androgen response in prostate cancers. EMBO J..

[87] Wilson S., Qi J., Filipp F.V. (2016). Refinement of the androgen response element based on ChIP-Seq in androgen-insensitive and androgen-responsive prostate cancer cell lines. Sci. Rep..

[88] Wang Q., Li W., Liu X.S., Carroll J.S., Janne O.A., Keeton E.K., Chinnaiyan A.M., Pienta K.J., Brown M. (2007). A hierarchical network of transcription factors governs androgen receptor-dependent prostate cancer growth. Mol. Cell.

[89] Witte T., Plass C., Gerhauser C. (2014). Pan-cancer patterns of DNA methylation. Genome Med..

[90] Bock C., Beerman I., Lien W.H., Smith Z.D., Gu H., Boyle P., Gnirke A., Fuchs E., Rossi D.J., Meissner A. (2012). DNA Methylation Dynamics during In Vivo Differentiation of Blood and Skin Stem Cells. Mol. Cell.

[91] Krum S.A., Miranda-Carboni G.A., Lupien M., Eeckhoute J., Carroll J.S., Brown M. (2008). Unique ERα Cistromes Control Cell Type-Specific Gene Regulation. Mol. Endocrinol..

[92] Ogoshi K., Hashimoto S., Nakatani Y., Qu W., Oshima K., Tokunaga K., Sugano S., Hattori M., Morishita S., Matsushima K. (2011). Genome-wide profiling of DNA methylation in human cancer cells. Genomics.

[93] Irizarry R.A., Ladd-Acosta C., Wen B., Wu Z., Montano C., Onyango P., Cui H., Gabo K., Rongione M., Webster M. (2009). The human colon cancer methylome shows similar hypo- and hypermethylation at conserved tissue-specific CpG island shores. Nat. Genet..

[94] Johnson K.C., Houseman E.A., King J.E., von Herrmann K.M., Fadul C.E., Christensen B.C. (2016). 5-Hydroxymethylcytosine localizes to enhancer elements and is associated with survival in glioblastoma patients. Nat. Commun..

[95] Bogdanovic O., Smits A.H., Mustienes E.D., Tena J.J., Ford E., Williams R., Senanayake U., Schultz M.D., Hontelez S., van Kruijsbergen I. (2016). Active DNA demethylation at enhancers during the vertebrate phylotypic period. Nat. Genet..

[96] Charlet J., Duymich C.E., Lay F.D., Mundbjerg K., Sorensen K.D., Liang G., Jones P.A. (2016). Bivalent Regions of Cytosine Methylation and H3K27 Acetylation Suggest an Active Role for DNA Methylation at Enhancers. Mol. Cell.

[97] Engreitz J.M., Haines J.E., Perez E.M., Munson G., Chen J., Kane M., McDonel P.E., Guttman M., Lander E.S. (2016). Local regulation of gene expression by lncRNA promoters, transcription and splicing. Nature.

[98] Ebisuya M., Yamamoto T., Nakajima M., Nishida E. (2008). Ripples from neighbouring transcription. Nat. Cell Biol..

[99] Kosak S.T., Scalzo D., Alworth S.V., Li F., Palmer S., Enver T., Lee J.S., Groudine M. (2007). Coordinate Gene Regulation During Hematopoiesis Is Related to Genomic Organization. PLoS Biol..

[100] Medvedeva Y.A., Khamis A.M., Kulakovskiy I.V., Ba-Alawi W., Bhuyan M.S., Kawaji H., Lassmann T., Harbers M., Forrest A.R., Bajic V.B. (2014). Effects of cytosine methylation on transcription factor binding sites. BMC Genom..

[101] Aran D., Hellman A. (2013). DNA methylation of transcriptional enhancers and cancer predisposition. Cell.

[102] Kim M.C., Kim N.Y., Seo Y.R., Kim Y. (2016). An Integrated Analysis of the Genome-Wide Profiles of DNA Methylation and mRNA Expression Defining the Side Population of a Human Malignant Mesothelioma Cell Line. J. Cancer.

[103] He D.X., Gu F., Gao F., Hao J.J., Gong D., Gu X.T., Mao A.Q., Jin J., Fu L., Ma X. (2016). Genome-wide profiles of methylation, microRNAs, and gene expression in chemoresistant breast cancer. Sci. Rep..

[104] Bjaanaes M.M., Fleischer T., Halvorsen A.R., Daunay A., Busato F., Solberg S., Jorgensen L., Kure E., Edvardsen H., Borresen-Dale A.L. (2016). Genome-wide DNA methylation analyses in lung adenocarcinomas: Association with EGFR, KRAS and TP53 mutation status, gene expression and prognosis. Mol. Oncol..

[105] Chen C., Peng H., Huang X., Zhao M., Li Z., Yin N., Wang X., Yu F., Yin B., Yuan Y. (2016). Genome-wide profiling of DNA methylation and gene expression in esophageal squamous cell carcinoma. Oncotarget.

[106] Singhal S.K., Usmani N., Michiels S., Metzger-Filho O., Saini K.S., Kovalchuk O., Parliament M. (2016). Towards understanding the breast cancer epigenome: A comparison of genome-wide DNA methylation and gene expression data. Oncotarget.

[107] Schroder F.H., Hugosson J., Roobol M.J., Tammela T.L., Ciatto S., Nelen V., Kwiatkowski M., Lujan M., Lilja H., Zappa M. (2009). Screening and prostate-cancer mortality in a randomized European study. N. Engl. J. Med..

[108] Andriole G.L., Crawford E.D., Grubb R.L., Buys S.S., Chia D., Church T.R., Fouad M.N., Gelmann E.P., Kvale P.A., Reding D.J. (2009). Mortality results from a randomized prostate-cancer screening trial. N. Engl. J. Med..

[109] Freedland S.J. (2011). Screening, risk assessment, and the approach to therapy in patients with prostate cancer. Cancer.

[110] Cookson M.S., Aus G., Burnett A.L., Canby-Hagino E.D., D’Amico A.V., Dmochowski R.R., Eton D.T., Forman J.D., Goldenberg S.L., Hernandez J. (2007). Variation in the definition of biochemical recurrence in patients treated for localized prostate cancer: The American Urological Association Prostate Guidelines for Localized Prostate Cancer Update Panel report and recommendations for a standard in the reporting of surgical outcomes. J. Urol..

[111] Yamoah K., Deville C., Vapiwala N., Spangler E., Zeigler-Johnson C.M., Malkowicz B., Lee D.I., Kattan M., Dicker A.P., Rebbeck T.R. (2015). African American men with low-grade prostate cancer have increased disease recurrence after prostatectomy compared with Caucasian men. Urol. Oncol..

[112] Ludwig M.S., Kuban D.A., Strom S.S., Du X.L., Lopez D.S., Yamal J.M. (2015). Assessing the Optimum Use of Androgen-Deprivation Therapy in High-Risk Prostate Cancer Patients Undergoing External Beam Radiation Therapy. Am. J. Mens. Health.

[113] Jones J., Grizzle W., Wang H., Yates C. (2013). MicroRNAs that affect prostate cancer: Emphasis on prostate cancer in African Americans. Biotechnol. Histochem..

[114] Powell I.J., Bollig-Fischer A. (2013). Minireview: The molecular and genomic basis for prostate cancer health disparities. Mol. Endocrinol..

[115] Drake B.F., Keane T.E., Mosley C.M., Adams S.A., Elder K.T., Modayil M.V., Ureda J.R., Hebert J.R. (2006). Prostate cancer disparities in South Carolina: Early detection, special programs, and descriptive epidemiology. J.S.C. Med. Assoc..

[116] Cooney K.A. (1998). Hereditary prostate cancer in African-American families. Semin. Urol. Oncol..

[117] Fraser M., Sabelnykova V.Y., Yamaguchi T.N., Heisler L.E., Livingstone J., Huang V., Shiah Y.J., Yousif F., Lin X., Masella A.P. (2017). Genomic hallmarks of localized, non-indolent prostate cancer. Nature.

[118] Li P.E., Nelson P.S. (2001). Prostate cancer genomics. Curr. Urol. Rep..

[119] Isaacs W.B. (1995). Molecular genetics of prostate cancer. Cancer Surv..

[120] Baca S.C., Prandi D., Lawrence M.S., Mosquera J.M., Romanel A., Drier Y., Park K., Kitabayashi N., MacDonald T.Y., Ghandi M. (2013). Punctuated evolution of prostate cancer genomes. Cell.

[121] Barbieri C.E., Baca S.C., Lawrence M.S., Demichelis F., Blattner M., Theurillat J.P., White T.A., Stojanov P., van Allen E., Stransky N. (2012). Exome sequencing identifies recurrent SPOP, FOXA1 and MED12 mutations in prostate cancer. Nat. Genet..

[122] Cancer Genome Atlas Research Network (2015). The Molecular Taxonomy of Primary Prostate Cancer. Cell.

[123] Taylor B.S., Schultz N., Hieronymus H., Gopalan A., Xiao Y., Carver B.S., Arora V.K., Kaushik P., Cerami E., Reva B. (2010). Integrative genomic profiling of human prostate cancer. Cancer Cell.

[124] Sandoval J., Esteller M. (2012). Cancer epigenomics: Beyond genomics. Curr. Opin. Genet. Dev..

[125] Varambally S., Dhanasekaran S.M., Zhou M., Barrette T.R., Kumar-Sinha C., Sanda M.G., Ghosh D., Pienta K.J., Sewalt R.G., Otte A.P. (2002). The polycomb group protein EZH2 is involved in progression of prostate cancer. Nature.

[126] Saramaki O.R., Tammela T.L., Martikainen P.M., Vessella R.L., Visakorpi T. (2006). The gene for polycomb group protein enhancer of zeste homolog 2 (EZH2) is amplified in late-stage prostate cancer. Genes Chromosom. Cancer.

[127] Ho V.T., Vanneman M., Kim H., Sasada T., Kang Y.J., Pasek M., Cutler C., Koreth J., Alyea E., Sarantopoulos S. (2009). Biologic activity of irradiated, autologous, GM-CSF-secreting leukemia cell vaccines early after allogeneic stem cell transplantation. Proc. Natl. Acad. Sci. USA.

[128] Borno S.T., Fischer A., Kerick M., Falth M., Laible M., Brase J.C., Kuner R., Dahl A., Grimm C., Sayanjali B. (2012). Genome-wide DNA methylation events in TMPRSS2-ERG fusion-negative prostate cancers implicate an EZH2-dependent mechanism with miR-26a hypermethylation. Cancer Discov..

[129] Kasinski A.L., Slack F.J. (2011). Epigenetics and genetics. MicroRNAs en route to the clinic: Progress in validating and targeting microRNAs for cancer therapy. Nat. Rev. Cancer.

[130] Singh P.K., Preus L., Hu Q., Yan L., Long M.D., Morrison C.D., Nesline M., Johnson C.S., Koochekpour S., Kohli M. (2014). Serum microRNA expression patterns that predict early treatment failure in prostate cancer patients. Oncotarget.

[131] Singh P.K., Campbell M.J. (2013). The Interactions of microRNA and Epigenetic Modifications in Prostate Cancer. Cancers.

[132] Massie C.E., Mills I.G., Lynch A.G. (2017). The importance of DNA methylation in prostate cancer development. J. Steroid Biochem. Mol. Biol..

[133] Campbell M.J., Park S., Uskokovic M.R., Dawson M.I., Koeffler H.P. (1998). Expression of retinoic acid receptor-beta sensitizes prostate cancer cells to growth inhibition mediated by combinations of retinoids and a 19-nor hexafluoride vitamin D3 analog. Endocrinology.

[134] Litovkin K., van Eynde A., Joniau S., Lerut E., Laenen A., Gevaert T., Gevaert O., Spahn M., Kneitz B., Gramme P. (2015). DNA Methylation-Guided Prediction of Clinical Failure in High-Risk Prostate Cancer. PLoS ONE.

[135] Haldrup C., Mundbjerg K., Vestergaard E.M., Lamy P., Wild P., Schulz W.A., Arsov C., Visakorpi T., Borre M., Hoyer S. (2013). DNA methylation signatures for prediction of biochemical recurrence after radical prostatectomy of clinically localized prostate cancer. J. Clin. Oncol..

[136] Wang Y., Jadhav R.R., Liu J., Wilson D., Chen Y., Thompson I.M., Troyer D.A., Hernandez J., Shi H., Leach R.J. (2016). Roles of Distal and Genic Methylation in the Development of Prostate Tumorigenesis Revealed by Genome-wide DNA Methylation Analysis. Sci. Rep..

[137] Li L., Li C., Mao H., Du Z., Chan W.Y., Murray P., Luo B., Chan A.T., Mok T.S., Chan F.K. (2016). Epigenetic inactivation of the CpG demethylase TET1 as a DNA methylation feedback loop in human cancers. Sci. Rep..

[138] Kinney S.R.M., Smiraglia D.J., James S.R., Moser M.T., Foster B.A., Karpf A.R. (2008). Stage-specific alterations of DNA methyltransferase expression, DNA hypermethylation, and DNA hypomethylation during prostate cancer progression in the transgenic adenocarcinoma of mouse prostate model. Mol. Cancer Res..

[139] Camoriano M., Kinney S.R., Moser M.T., Foster B.A., Mohler J.L., Trump D.L., Karpf A.R., Smiraglia D.J. (2008). Phenotype-specific CpG island methylation events in a murine model of prostate cancer. Cancer Res..

[140] Morey S.R., Smiraglia D.J., James S.R., Yu J., Moser M.T., Foster B.A., Karpf A.R. (2006). DNA methylation pathway alterations in an autochthonous murine model of prostate cancer. Cancer Res..

[141] Spans L., van den Broeck T., Smeets E., Prekovic S., Thienpont B., Lambrechts D., Karnes R.J., Erho N., Alshalalfa M., Davicioni E. (2016). Genomic and epigenomic analysis of high-risk prostate cancer reveals changes in hydroxymethylation and TET1. Oncotarget.

[142] Yang Y.A., Zhao J.C., Fong K.W., Kim J., Li S., Song C., Song B., Zheng B., He C., Yu J. (2016). FOXA1 potentiates lineage-specific enhancer activation through modulating TET1 expression and function. Nucleic Acids Res..

[143] Labbe D.P., Zadra G., Ebot E.M., Mucci L.A., Kantoff P.W., Loda M., Brown M. (2015). Role of diet in prostate cancer: The epigenetic link. Oncogene.

[144] Bistulfi G., Affronti H.C., Foster B.A., Karasik E., Gillard B., Morrison C., Mohler J., Phillips J.G., Smiraglia D.J. (2016). The essential role of methylthioadenosine phosphorylase in prostate cancer. Oncotarget.

[145] Bistulfi G., Vandette E., Matsui S., Smiraglia D.J. (2010). Mild folate deficiency induces genetic and epigenetic instability and phenotype changes in prostate cancer cells. BMC Biol..

[146] Bistulfi G., Diegelman P., Foster B.A., Kramer D.L., Porter C.W., Smiraglia D.J. (2009). Polyamine biosynthesis impacts cellular folate requirements necessary to maintain *S*-adenosylmethionine and nucleotide pools. FASEB J..

[147] Shabbeer S., Williams S.A., Simons B.W., Herman J.G., Carducci M.A. (2012). Progression of prostate carcinogenesis and dietary methyl donors: Temporal dependence. Cancer Prev. Res. (Phila).

[148] Kutmon M., Riutta A., Nunes N., Hanspers K., Willighagen E.L., Bohler A., Melius J., Waagmeester A., Sinha S.R., Miller R. (2016). WikiPathways: Capturing the full diversity of pathway knowledge. Nucleic Acids Res..

[149] Pundir S., Magrane M., Martin M.J., O’Donovan C., UniProt C. (2015). Searching and Navigating UniProt Databases. Curr. Protoc. Bioinform..

[150] Long M.D., Campbell M.J. (2015). Pan-cancer analyses of the nuclear receptor superfamily. Nucl. Recept. Res..

[151] Doig C.L., Singh P.K., Dhiman V.K., Thorne J.L., Battaglia S., Sobolewski M., Maguire O., O’Neill L.P., Turner B.M., McCabe C.J. (2013). Recruitment of NCOR1 to VDR target genes is enhanced in prostate cancer cells and associates with altered DNA methylation patterns. Carcinogenesis.

[152] Larkin S.E., Holmes S., Cree I.A., Walker T., Basketter V., Bickers B., Harris S., Garbis S.D., Townsend P.A., Aukim-Hastie C. (2012). Identification of markers of prostate cancer progression using candidate gene expression. Br. J. Cancer.

[153] Schmidt T., Leha A., Salinas-Riester G. (2016). Treatment of prostate cancer cells with *S*-adenosylmethionine leads to genome-wide alterations in transcription profiles. Gene.

[154] Shukeir N., Stefanska B., Parashar S., Chik F., Arakelian A., Szyf M., Rabbani S.A. (2015). Pharmacological methyl group donors block skeletal metastasis in vitro and in vivo. Br. J. Pharmacol..

[155] Chen M., Huang Y.L., Huang Y.C., Shui I.M., Giovannucci E., Chen Y.C., Chen Y.M. (2014). Genetic Polymorphisms of the Glycine *N*-Methyltransferase and Prostate Cancer risk in the Health Professionals Follow-Up Study. PLoS ONE.

[156] Li W., Liu X., Wang W., Sun H., Hu Y., Lei H., Liu G., Gao Y. (2008). Effects of antisense RNA targeting of ODC and AdoMetDC on the synthesis of polyamine synthesis and cell growth in prostate cancer cells using a prostatic androgen-dependent promoter in adenovirus. Prostate.

[157] Kee K., Vujcic S., Merali S., Diegelman P., Kisiel N., Powell C.T., Kramer D.L., Porter C.W. (2004). Metabolic and antiproliferative consequences of activated polyamine catabolism in LNCaP prostate carcinoma cells. J. Biol. Chem..

[158] Mamont P.S., Danzin C., Wagner J., Siat M., Joder-Ohlenbusch A.M., Claverie N. (1982). Accumulation of Decarboxylated *S*-adenosyl-l-Methionine in Mammalian Cells as a Consequence of the Inhibition of Putrescine Biosynthesis. Eur. J. Biochem..

